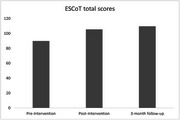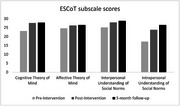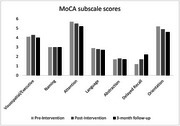# A novel social cognition intervention for people with mild cognitive impairment or early stage dementia: Pilot study results

**DOI:** 10.1002/alz70858_106202

**Published:** 2025-12-24

**Authors:** Suraj Samtani, Niki McDonagh, Anne‐Nicole Casey, Perminder S. Sachdev, Henry Brodaty, Julie D Henry

**Affiliations:** ^1^ Centre for Healthy Brain Ageing (CHeBA), UNSW Sydney, Sydney, NSW, Australia; ^2^ University of Queensland, Brisbane, QLD, Australia

## Abstract

**Background:**

Social cognition underpins our ability to maintain social connections, which in turn can reduce our risk of dementia. Existing social cognitive interventions for people living with mild cognitive impairment or early stage dementia involve either informal social gatherings or target only a single social cognitive skill, such as emotion recognition. There are therefore currently no interventions that target the broad range of social cognitive skills involved in maintaining social connections for people living with mild cognitive impairment or early‐stage dementia.

**Method:**

A novel co‐designed social cognitive skills intervention was delivered online to 10 people (6 females and 4 males; *M*_age_ = 69 years, _range_ = 58‐82 years) with mild cognitive impairment (*n* = 7) and early‐stage self‐reported dementia (*n* = 3). The intervention was delivered by a psychologist via videocalls with accompany slides and a participant booklet in groups of 3‐4 participants each, for five 2‐hour weekly sessions. Sessions included: awareness of everyday communication skills; development of a personal action plan and reading non‐verbal communication; managing sensory and cognitive challenges; real world interactions including assertiveness, confidence, building and maintaining good relationships; program review; and revisiting the personal action plan. Social cognition was measured using the Edinburgh Social Cognition Test (ESCoT). Secondary outcomes included cognition (MoCA), social frailty (social frailty index and social frailty scale), loneliness (UCLA loneliness scale), mental health (Geriatric Depression and Anxiety Scales), and quality of life (AQoL‐6D). Data were collected via online interviews at pre‐intervention, post‐intervention, and at 3‐month follow‐up.

**Result:**

ESCoT scores improved 17% at the end of the intervention, with further improvement at 3‐months’ follow‐up (21.8%). Delayed recall scores on the MoCA also improved by 83% by 3‐month follow‐up. Mental health symptoms and loneliness scores showed a floor effect. Participants reported finding the online intervention easy to access.

**Conclusion:**

A novel online group intervention for social cognitive skills showed promising results in a pilot study with people with mild cognitive impairment and early‐stage dementia. A randomised controlled trial with a larger sample is required to test the effectiveness of the intervention in improving social cognition.